# Myricetin Attenuated Diabetes-Associated Kidney Injuries and Dysfunction *via* Regulating Nuclear Factor (Erythroid Derived 2)-Like 2 and Nuclear Factor-κB Signaling

**DOI:** 10.3389/fphar.2019.00647

**Published:** 2019-06-11

**Authors:** Zi-Jun Yang, Hong-Ru Wang, Yu-Iin Wang, Zi-Han Zhai, Liu-Wei Wang, Liang Li, Cheng Zhang, Lin Tang

**Affiliations:** Department of Nephropathy, The First Affiliated Hospital of Zhengzhou University, Zhengzhou, China

**Keywords:** myricetin, diabetic nephropathy, nuclear factor (erythroid derived 2)-like 2, IκB/nuclear factor-κB (P65), effects

## Abstract

**Background/Aims:** Previous studies have suggested that myricetin (Myr) could promote the expression and nuclear translocation of nuclear factor (erythroid-derived 2)-like (Nrf2). This study aimed to investigate whether Myr could attenuate diabetes-associated kidney injuries and dysfunction in wild-type (WT) and Nrf2 knockdown (Nrf2-KD) mice.

**Methods:** Lentivirus-mediated Nrf2-KD and WT mice were used to establish type 1 diabetes mellitus (DM) by streptozotocin (STZ) injection. WT and Nrf2-KD mice were then randomly allocated into four groups: control (CON), Myr, STZ, and STZ + Myr. Myr (100 mg/kg/day) or vehicle was administered for 6 months. Kidneys were harvested and weighed at the end of the experiment. Hematoxylin and eosin staining and Masson’s trichrome staining were used to assess the morphology and fibrosis of the kidneys, respectively. Urinary albumin-to-creatinine ratio was used to test renal function. Western blotting was performed to determine oxidative-stress- or inflammation-associated signaling pathways. Real-time polymerase chain reaction (RT-PCR) was performed to detect the expression of fibrosis or inflammatory cytokines at the message Ribonucleic Acid (mRNA) level.

**Results:** In WT mice, Myr alleviated DM-induced renal dysfunction, fibrosis, and oxidative damage and enhanced the expression of Nrf2 and its downstream genes. After knockdown of Nrf2, Myr treatment partially but significantly mitigated DM-induced renal dysfunction and fibrosis, which might be associated with inhibition of the I-kappa-B (IκB)/nuclear factor-κB (NF-κB) (P65) signaling pathway.

**Conclusions:** This study showed that Myr prevented DM-associated decreased expression of Nrf2 and inhibited IκB/NF-κB (P65) signaling pathway. Moreover, inhibition of IκB/NF-κB (P65) signaling pathway is independent of the regulation of Nrf2. Thus, Myr could be a potential treatment for preventing the development and progression of DM-associated kidney injuries and dysfunction.

## Introduction

Diabetic nephropathy (DN) is one of the major complications of diabetes mellitus (DM). Approximately 30–40% of patients with diabetes develop DN, and one-third of them progress to end-stage renal disease (ESRD) (Tesch, 2017). There is currently no effective therapy available for ESRD patients except for dialysis and transplantation. However, patients undergoing dialysis still have a high mortality rate of 20% per year (DeFronzo et al., 2017), and the shortage of donor kidneys largely restricts the number of transplantations (DeFronzo et al., 2017). At present, strategies for DN treatment mainly focus on glycemic and blood pressure control, and there is no effective therapy for blocking the progression of renal failure initiated through DN (Lacava et al., 2017). Because of the high morbidity of DN and the rapidly increasing numbers of diabetic patients worldwide, new effective treatments are urgently needed to counter DM-associated kidney injuries and dysfunction.

Oxidative stress and inflammation have been widely accepted as two main causes of DM-associated kidney injuries and dysfunction (Jha et al., 2016; Li et al., 2017). Accumulating evidence indicates that overproduction of reactive oxygen species (ROS) is a common and pivotal characteristic associated with DM-associated kidney injuries and dysfunction (Jha et al., 2016). Excessive ROS in podocytes and mesangial or endothelial cells could activate the nuclear factor (NF)-κB signaling pathway, resulting in the accumulation of inflammatory factors such as tumor necrosis factor (TNF)-α, interleukin (IL)-1β, IL-6, and monocyte chemoattractant protein (MCP)-1. Inflammation can in turn lead to tissue injury and further ROS accumulation. Indeed, oxidative stress and inflammation are reciprocal causes in the development of DM-associated kidney injuries and dysfunction. Thus, strategies aimed at reducing oxidative stress and inflammation may be potential treatments for DM-associated kidney injuries and dysfunction.

Myricetin (Myr), which is widely found in most berries, vegetables, and various medicinal herbs, has been suggested to possess potent antioxidative and anti-inflammatory capabilities. Microarray and pathway analysis indicated that Myr might particularly inhibit the degradation of nuclear factor (erythroid derived 2)-like 2 (Nrf2) and promote nuclear translocation (Qin et al., 2013). Nrf2 has been demonstrated to be a transcription factor that prevents ROS-induced tissue injury and shows beneficial effects in a variety of kidney diseases, including acute kidney disease (Shelton et al., 2013), chronic kidney disease (Hasegawa et al., 2017), DN (Wu et al., 2016), and hypertension-associated kidney disease (Chang et al., 2013). Besides the protective effects against oxidative stress through Nrf2 activation, Myr can also protect the kidney from cisplatin-induced toxicity partly by decreasing the number of inflammatory mediators, including TNF-α and IL-6 (Hassan et al., 2017). Its anti-inflammatory function might be associated with the inhibition of NF-κB and mitogen-activated protein kinase (MAPK) signaling pathways (Fu et al., 2013).

In this study, we aimed to discover whether Myr could attenuate DM-associated kidney injuries and dysfunction in experimental DM mouse model induced by five consecutive injections of low-dose streptozotocin (STZ). We also tried to investigate whether the renal protective effects of Myr were associated with Nrf2 activation and the inhibition of inflammation.

## Materials and Methods

### Animals

Mice (C57BL/6, 9–10 weeks, 24.5–25.5 g) were purchased from the Laboratory of Animal Science, Chinese Academy Medical Sciences and Peking Union Medical College (Beijing, China). All animals were housed at the Research Institute of Zhengzhou University (Zhengzhou, China) at 22°C with a 12-h light/dark cycle and free access to food and water. All animals were adapted to the environment for 1 week before the experimental procedures. All experimental procedures were approved by the institutional guidelines of the Animal Care and Use Committee of The First Affiliated Hospital of Zhengzhou University, which is compliant with the Guidelines for the Care of Laboratory Animals established by the National Institutes of Health (Bethesda, MD).

### Establishment of Diabetes Mellitus Mouse Model

The DM mouse model was established according to published protocols (Alghamdi et al., 2018; Guo et al., 2018). Briefly, to establish the DM mouse model, wild-type mice were injected with STZ (Sigma-Aldrich, St. Louis, MO) intraperitoneally at a dose of 50 mg/kg/day for 5 consecutive days. STZ was dissolved in 0.1 M sodium citrate buffer (pH = 4.5). Mice in the control (Con) group received injections of the same volume of sodium citrate buffer. Five days after the last injection, mice with increased blood glucose levels of ≥250 mg/dL were defined as having DM. Mice were then randomly allocated into four groups: CON (*n* = 12), Myr (*n* = 12), STZ (*n* = 20), and STZ + Myr (*n* = 20) The dose of Myr was chosen based on a previous study and was orally administered twice a day (total 100 mg/kg/day) (Zhang et al., 2016; Geng et al., 2017). Myr was dissolved in double-distilled water. Both Con and diabetic mice received Myr for 6 months. At the end of the study, mice were euthanized, and the kidneys were harvested for the following studies.

### Generation of Short-Hairpin RNA-Expression Lentivirus for Nuclear Factor (Erythroid Derived 2)-Like 2 Knockdown

Small interfering RNA target sites for Nrf2 ([mouse, silence of nuclear factor erythroid-2-related factor 2 (siNrf2)]) were designed according to a published database (Yuan et al., 2004). A scrambled sequence was prepared as a control. Short-hairpin RNA (shRNA) expression for Nrf2 or the scrambled sequence was produced according to published protocols (Naito and Ui-Tei, 2013). Lentivirus samples were set up at a titer of 8 × 10^8^ transfection units (TU)/mL and stored at −80°C for the following experiments (Nam et al., 2015). To knock down Nrf2 *in vivo*, C57 mice were randomly divided into four groups and injected with corresponding lentivirus shRNA or scrambled sequences *via* tail vein injection at the age of 6 weeks. Lentivirus was injected at 3-day intervals for three consecutive injections (Nam et al., 2015). To maintain effective expression of shRNA, the injections were repeated every 4 weeks. Each injection was performed with lentivirus at a dose of 4 × 10^8^ TU. After the first three consecutive injections, mice were treated with STZ and Myr according to the method described in Establishment of Diabetes Mellitus Mouse Model. Mice were randomly allocated into four groups: CON (*n* = 12), siNrf2 (*n* = 12), siNrf2 + STZ (*n* = 12), and siNrf2 + STZ + Myr (*n* = 12).

### Western Blotting

Protein samples were extracted from the kidneys. The lysates (50 µg) were separated by sodium dodecyl sulfate–polyacrylamide gel electrophoresis with a 10% (wt/vol) acrylamide gel and transferred to polyvinylidene fluoride membranes. After blocking with 10% milk and incubating with primary and secondary antibodies, the blots were visualized by enhanced chemiluminescence. The intensity of each band was quantified by densitometry with Image Lab 5.2.1 and normalized to glyceraldehyde-3-phosphate dehydrogenase (GAPDH) before relative quantification. The primary antibodies used in our study included Nrf2 (ab62352, Abcam, Cambridge, UK), heme oxygenase 1 (HO-1; ab13248, Abcam), GAPDH (60004-1-Ig, Proteintech, Wuhan, China), Histone 3 (ab5176, Abcam), phosphorylated (p)-I-kappa-B-alpha (IκBα) (#2859, Cell Signaling Technology, Danvers, MA), IκBα (#4812, Cell Signaling Technology), p-P65 (BS4135, Bioworld, Dublin, OH), p65 (BS3648, Bioworld), transforming growth factor (TGF)-β1 (ab92486, Abcam), p-Smad1/5 (#9516, Cell Signaling Technology), p-Smad2 (#18338, Cell Signaling Technology), IL-6 (#12912, Cell Signaling Technology), and TNF-α (ab34674, Abcam).

### Real-Time Polymerase Chain Reaction

Total RNA was extracted from the kidneys by TRIzol reagent (Invitrogen, Carlsbad, CA) according to the manufacturer’s instructions. RNA sample (2 µg) was reverse transcribed to cDNA with the First Strand cDNA Synthesis Kit. Each sample was subject to duplicate examinations in triplicate. GAPDH was used as an endogenous control for relative quantification.

### Histology Examination

Kidney tissues were fixed in formalin, dehydrated, embedded in paraffin, and cut into 5-μm-thick sections. The sections were stained with hematoxylin and eosin (H&E) staining or Masson trichrome staining (MTS). A light microscope was used to acquire images, and MTS images were quantified using image-pro-plus (IPP) software.

### Urinary Albumin-to-Creatinine Ratio

Urinary albumin and urinary creatinine levels were tested with kits (Bethyl Laboratories, Inc., Montgomery, TX) according to the manufacturer’s instructions. Urinary albumin-to-creatinine ratio (UACR) was calculated according to the formula: UACR = urinary albumin/urinary creatinine (μg/mg).

### Statistical Analysis

Data were derived from at least 10 mice for each group and presented as the mean ± standard deviation (SD). Statistical product and service solutions (SPSS) version 19.0 software (SPSS Inc., Chicago, IL) was used to analyze the statistical significance of the results. One-way analysis of variance followed by Tukey’s *post hoc* test was conducted to compare the differences between the groups. Differences were considered significant when *p* < 0.05.

## Results

### Myricetin Treatment Prevented Kidney Injuries Induced by Diabetes Mellitus

**Figure 1:**
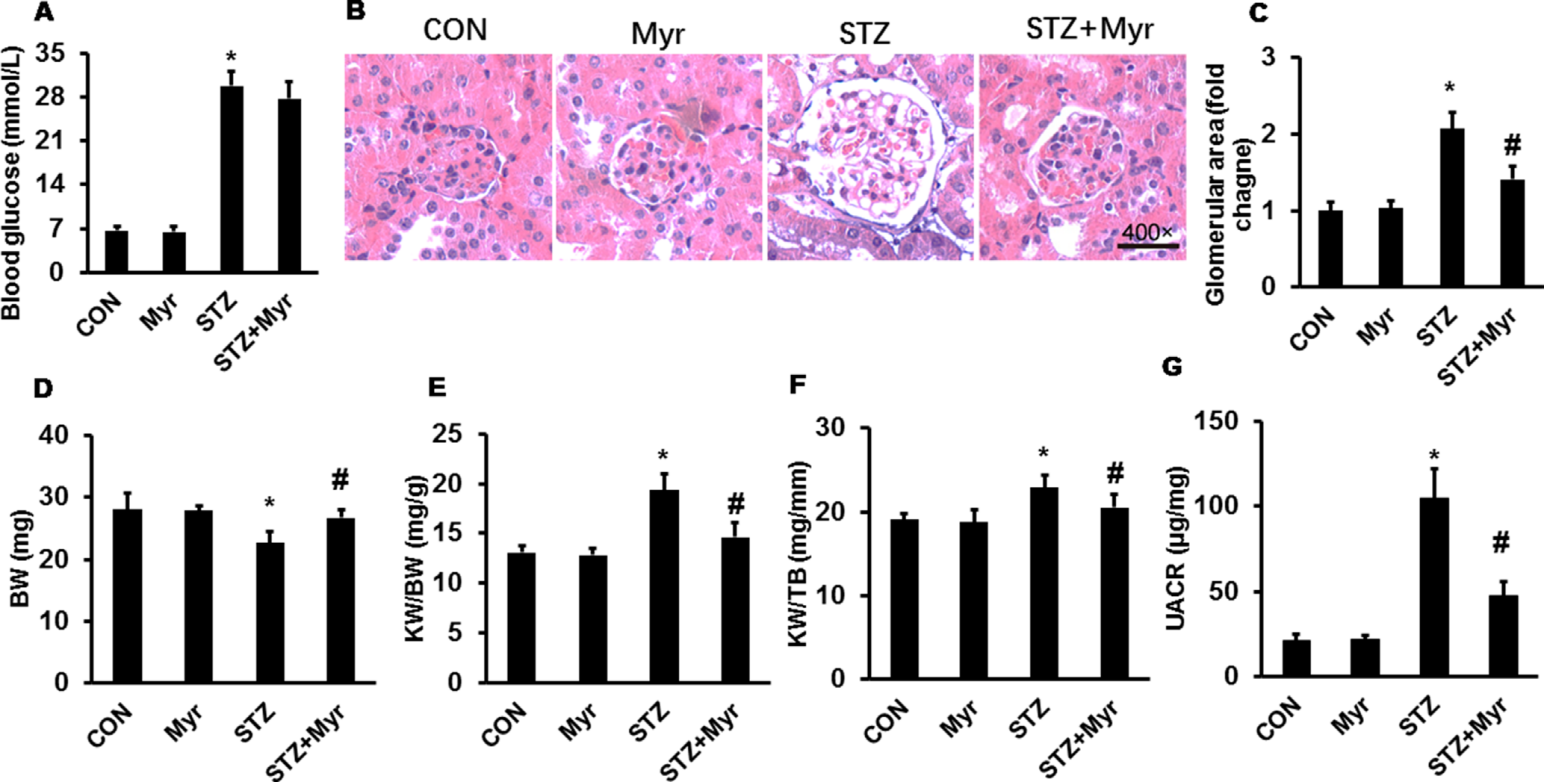
Myricetin treatment attenuated streptozotocin (STZ)-induced diabetic nephropathy. **(A)** Blood glucose (*n* ≥ 12); **(B)** representative images (400×) of hematoxylin and eosin (H&E) staining of kidneys (*n* = 6); **(C)** calculated glomerular area; **(D)** body weight (BW) (*n* ≥ 12); **(E)** kidney weight (KW)/BW ratio (*n* ≥ 12); **(F)** KW/tibia length (TB) ratio (*n* ≥ 12); **(G)** urinary albumin-to-creatinine ratio (UACR) (*n* ≥ 12). Data are presented as the mean ± SD. *p < 0.05 vs. the Con group, *^#^* *p* < 0.05 vs. the STZ group.

After 5 consecutive days of STZ injections, mice developed hyperglycemia; no significant differences were found for either UACR or body weight (BW) among the different groups (**Supplementary Table 2**). Myr seemed to be unable to reduce blood glucose levels after 6 months of treatment (**Figure 1A**). As shown in **Figure 1B and C**, the glomerular area was significantly enlarged in the STZ group, and Myr treatment clearly prevented this change. Meanwhile, we calculated BW and kidney weight (KW)/BW ratios and KW/tibia length (TB) ratios. STZ-induced DM caused a decrease in BW but increase in KW/BW and KW/TB ratios, while Myr treatment effectively blocked these changes (**Figure 1D–F**). As an important index for monitoring renal function, UACR was determined at the end of the study. The results revealed that STZ injections caused a much higher UACR compared with the Con group, and Myr treatment prevented this change (**Figure 1G**). In this part of investigation, no animals were lost from any of experimental groups prior to the 6 months end point. Taken together, these results suggest that Myr could protect against STZ-induced renal injuries.

### Myricetin Alleviated Oxidative Stress by Regulating the Nuclear Factor (Erythroid Derived 2)-Like 2/heme oxygenase-1 (HO-1) Pathway

**Figure 2:**
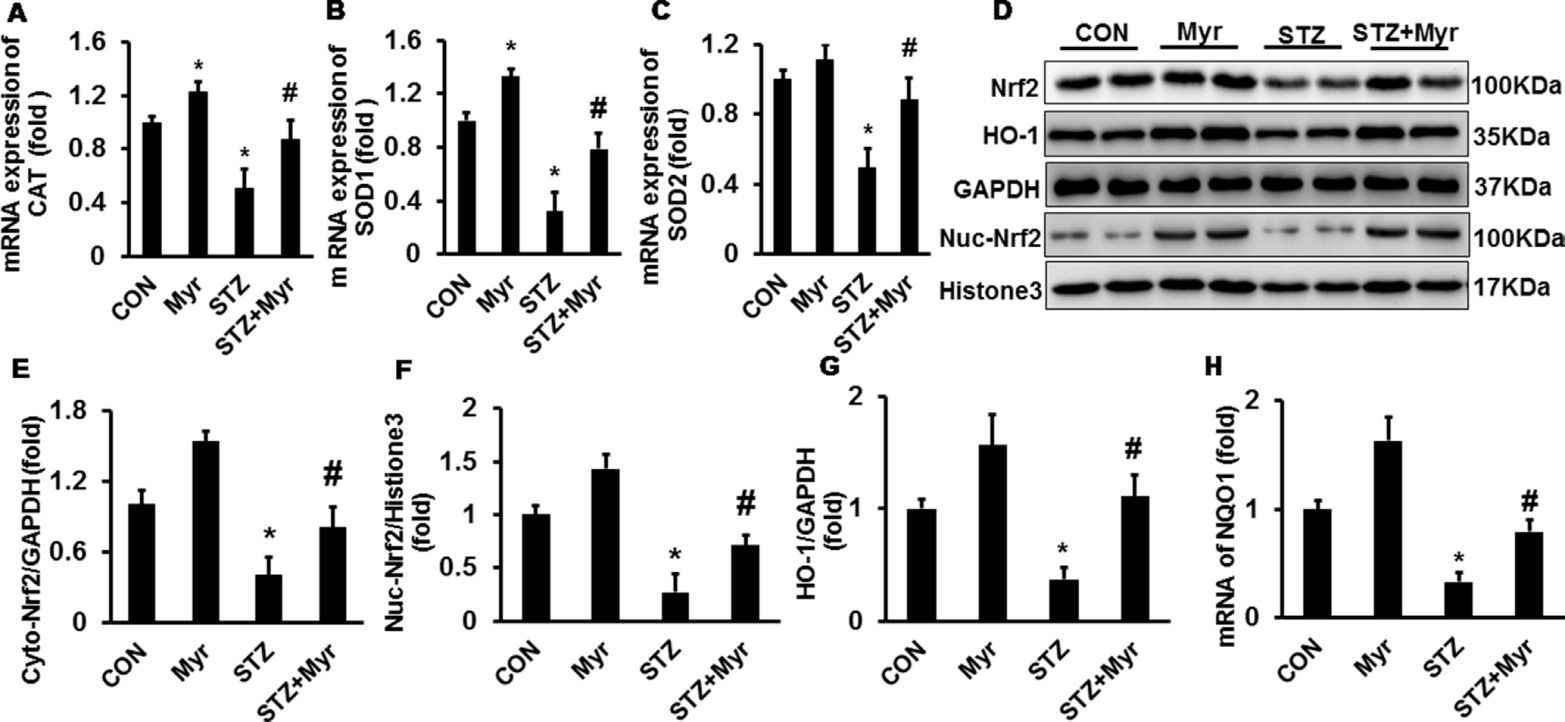
Myricetin alleviated diabetes mellitus (DM)-associated oxidative stress in mouse kidneys. **(A**–**C)** mRNA levels of CAT, SOD1, and SOD2 (*n* = 6); **(D)** representative blots of nuclear factor (erythroid-derived 2)-like (Nrf2), heme oxygenase 1, GAPDH, Nuc-Nrf2, and Histone3 (*n* = 6); **(E**–**G)** calculated protein expression levels (*n* = 6); **(H)** mRNA expression of NQO1 (*n* = 6). Data are presented as the mean ± SD. *p < 0.05 vs. the Con group, *^#^* *p* < 0.05 vs. the STZ group.

As shown in **Figure 2A–C**, the mRNA expression of antioxidative stress proteins, including catalase (CAT), superoxide dismutase 1 (SOD1), and superoxide dismutase 2 (SOD2), was downregulated in DM mice kidneys, and treatment with Myr significantly inhibited the downregulation of these markers (**Figure 2A–C**). These data strongly indicated the potent antioxidative ability of Myr in DM; thus, we further explored the underlying mechanism. Western blotting showed that Myr could promote the expression and nuclear accumulation of Nrf2 at baseline (**Figure 2D–F**). In the STZ group, the expression and nuclear translocation levels of Nrf2 were significantly decreased (**Figure 2D–F**), and treatment with Myr prevented this change (**Figure 2D–F**). Downstream of Nrf2, both HO-1 and NAD (P)H Dehydrogenase, Quinone 1 (NQO1) were blunted in the DM group at the protein and mRNA levels, respectively, while treatment with Myr recovered the expression of these antioxidant genes (**Figure 2G and H**).

### Myricetin Attenuated the Inflammatory Response *via* the IκBα/nuclear Factor-κB Pathway

**Figure 3:**
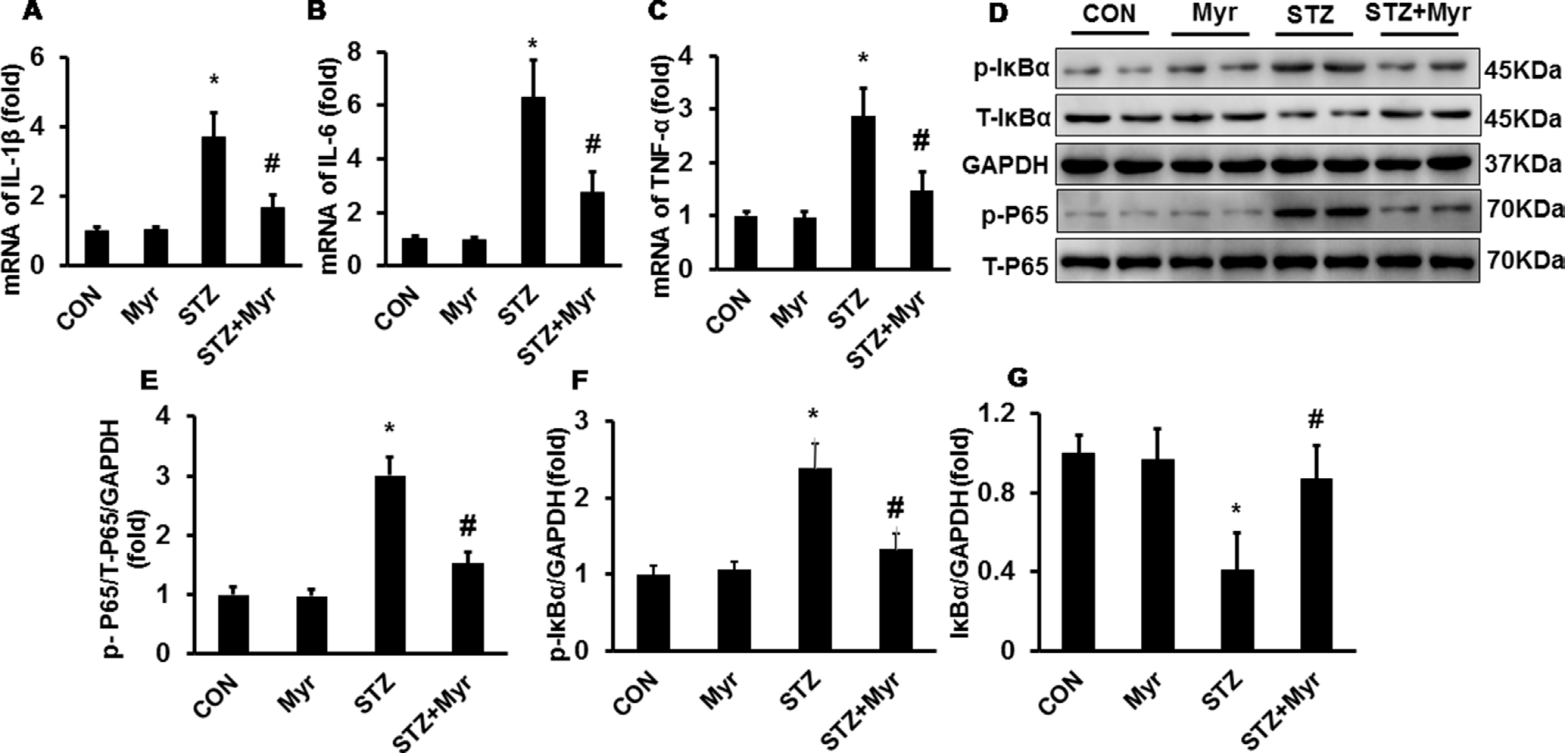
Myricetin reduced the DM-associated inflammatory response in mouse kidneys. **(A**–**C)** mRNA levels of interleukin (IL)-1β, IL-6, and tumor necrosis factor (TNF)-α (*n* = 6); **(D)** representative blots of phosphorylated (p)-IκB, IκB, GAPDH, p-P65, and P65 (*n* = 6); **(E**–**G)** calculated protein expression levels (*n* = 6). *p < 0.05 vs. the Con group, *^#^* *p* < 0.05 vs. the STZ group.

The levels of inflammatory markers including IL-1β, IL-6, and TNF-α were determined by RT-PCR. As shown in **Figure 3A–C**, DM increased the expression of IL-1β, IL-6, and TNF-α compared with that in the Con and Myr groups, while Myr treatment significantly blunted the expression of these inflammatory factors. We then examined IκB/p65 signaling, which has been demonstrated to regulate the expression of these inflammatory factors. DM significantly induced the phosphorylation of IκB, resulting in the degradation of total IκB and the phosphorylation of p65 (**Figure 3D–G**), while treatment with Myr inhibited the phosphorylation of IκB and p65, which led to inactivation of the IκB/NF-κB pathway (**Figure 3D–G**).

### Myricetin Protected Against Diabetes Mellitus-Induced Renal Fibrosis

**Figure 4:**
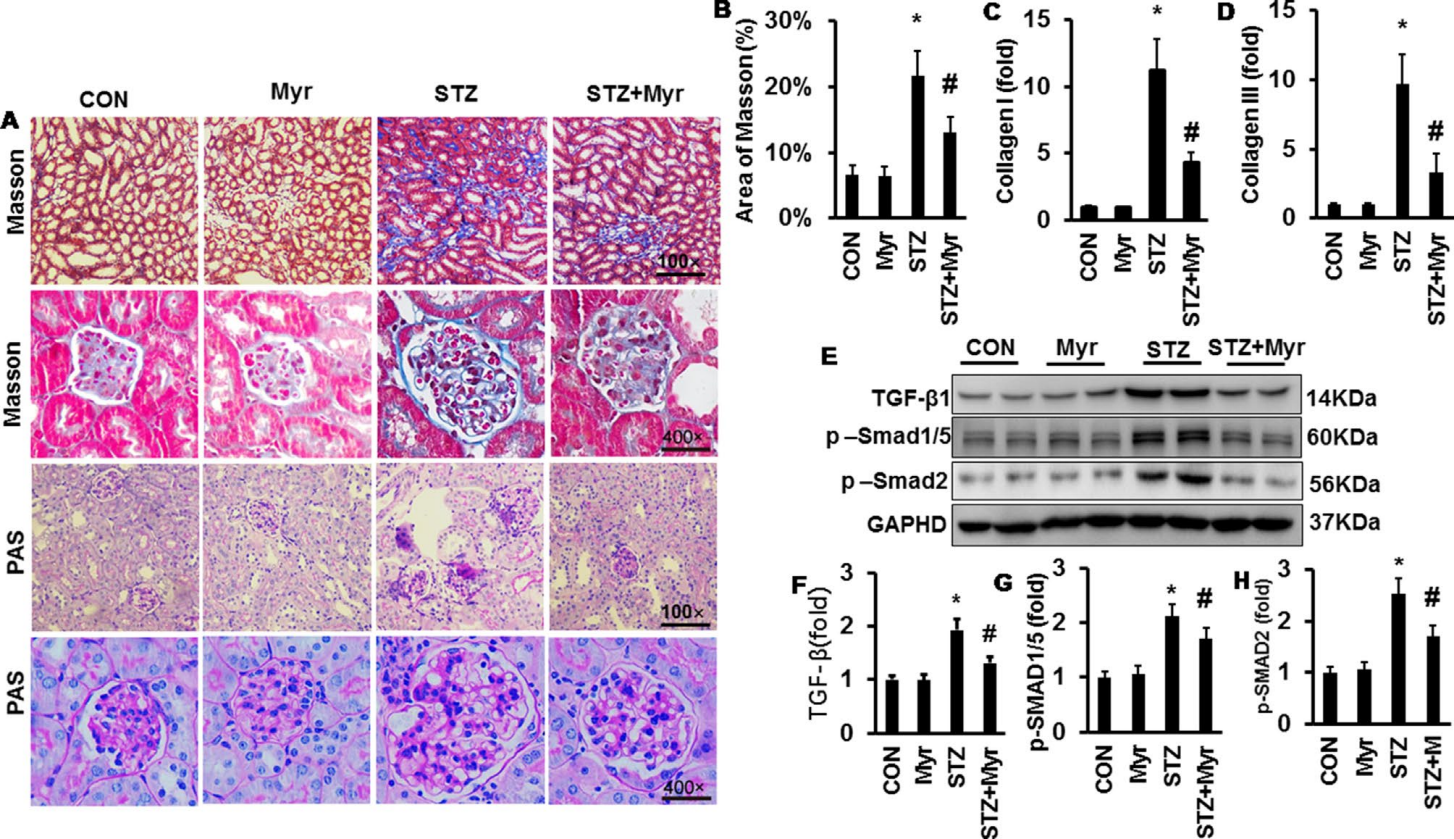
Myricetin prevented DM-induced fibrosis in mouse kidney. **(A)** Masson and periodic acid–Schiff (PAS) staining of kidneys (*n* = 6); **(B)** calculated positive area of Masson staining (*n* = 6); **(C** and **D)** mRNA level of collagen I and III (*n* = 6); **(E)** representative blots of transforming growth factor (TGF)-β, p-Smad1/5, Smad2, and GAPDH (*n* = 6); **(F**–**H)** calculated protein expression levels (*n* = 6); *p < 0.05 vs. the Con group, *^#^* *p* < 0.05 vs. the STZ group.

To investigate the effect of Myr on DM-induced renal fibrosis, MTS was performed (**Figure 4A**). Diabetic kidneys showed visible fibrosis with a positive area as high as about 22%; however, Myr markedly prevented kidney fibrosis (**Figure 4A and B**). Both collagen I and collagen III, two representative fibrosis indexes, were significantly increased in diabetic kidneys (**Figure 4C and D**) and blunted by Myr treatment (**Figure 4C and D**). The TGF-β/Smad signaling pathway is one of the most important pathways in regulating fibrosis. DM induced the activation of the TGF-β/Smad signaling pathway as evidenced by the overproduction of TGF-β hyperphosphorylation of Smad1/5 and Smad2 (**Figure 4E–H**), while Myr treatment significantly blunted these changes (**Figure 4E–H**). Periodic acid–Schiff staining was conducted to show glycogen deposition in mouse kidneys. In the diabetic mouse kidneys, glycogen was significantly deposited and white vacuoles were increased compared with those in the Con group (**Figure 4A**). The glycogen deposition and white vacuoles were significantly reduced in the kidneys after Myr treatment (**Figure 4A**).

### Myricetin Retained Partial Protection Against Diabetes Mellitus-Associated Renal Injury and Fibrosis after Nuclear Factor (Erythroid Derived 2)-Like 2 Knockdown *In Vivo*

**Figure 5:**
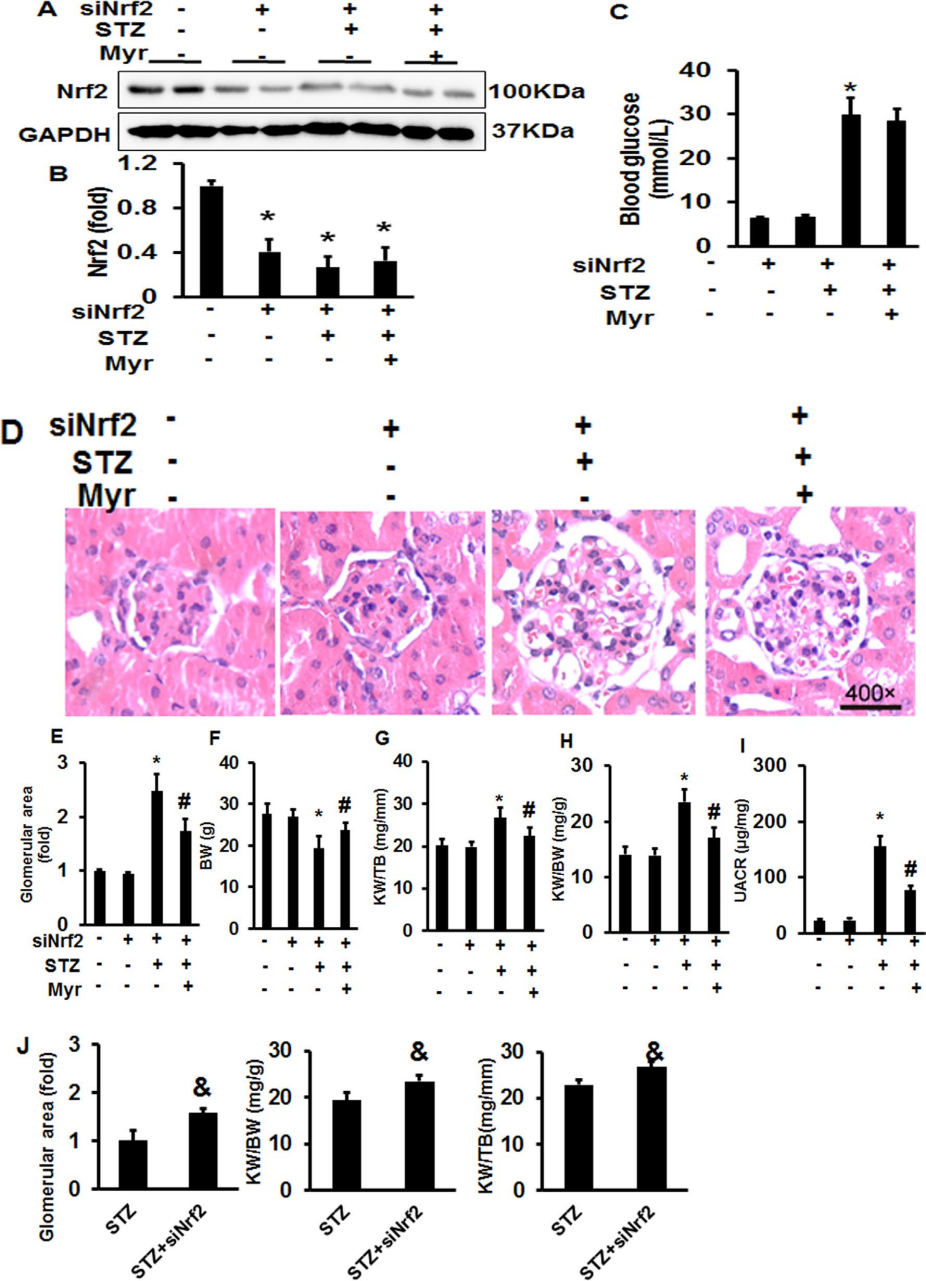
Myricetin retained partial protection against DM-associated renal injury after Nrf2 knockdown *in vivo*. **(A)** Representative blots of Nrf-2 and GAPDH (*n* = 6); **(B)** calculated protein expression levels of Nrf2 after normalized to GAPDH (*n* = 6); **(C)** blood glucose (*n* = 12); **(D)** representative images (400×) of H&E staining of kidneys (*n* = 6); **(E)** calculated glomerular area (*n* = 12); **(F)** BW (*n* = 12); **(G)** KW/BW ratio (*n* = 12); **(H)** KW/TB ratio (*n* = 12); **(I)** UACR (*n* = 12); **(J)** silence of Nrf2 exacerbated STZ-induced morphological change of mouse kidney. Data are presented as the mean ± SD. *p < 0.05 vs. the Con group, *^#^* *p* < 0.05 vs. the siNrf2+STZ group, ^&^ *p* < 0.05 vs. the STZ group.

**Figure 6:**
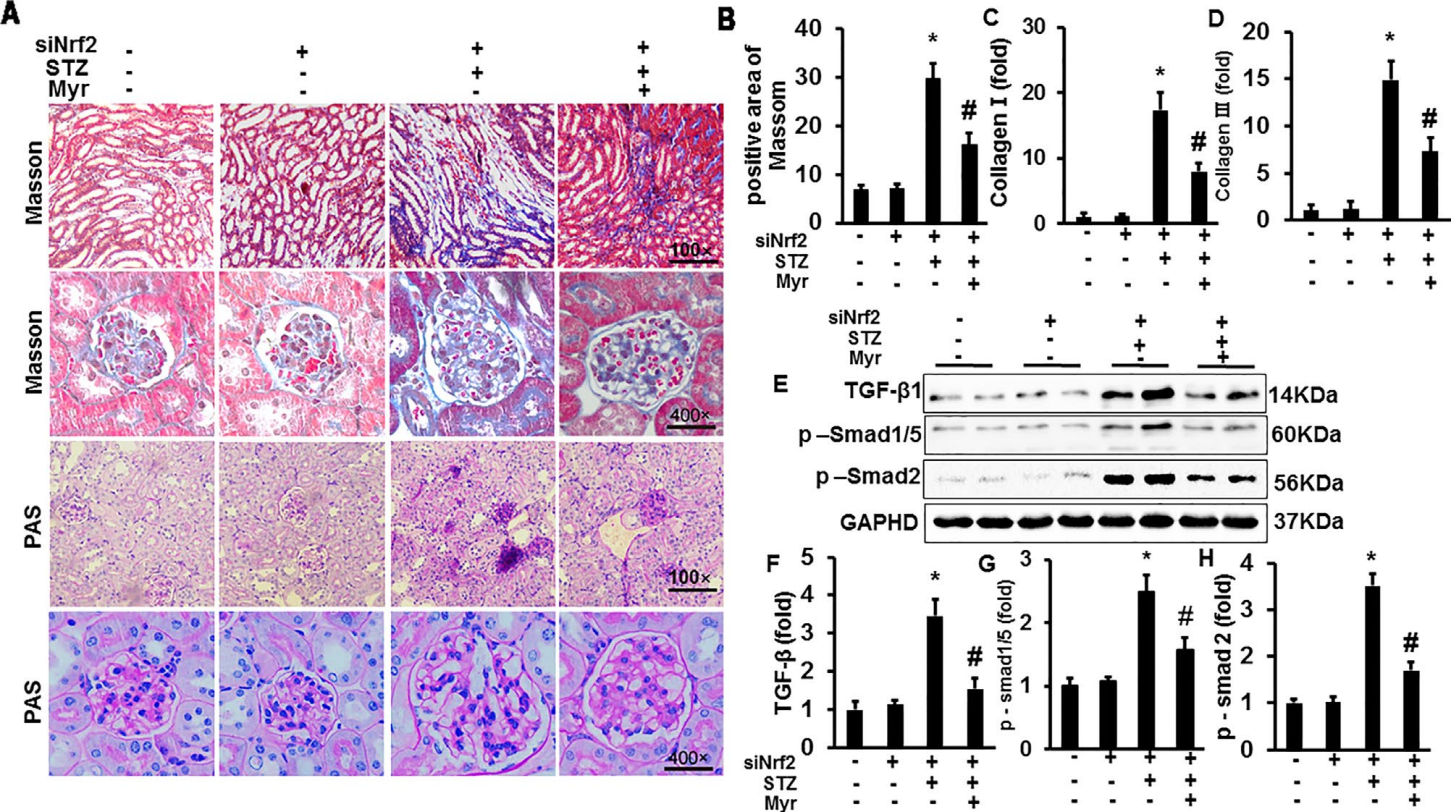
Myricetin retained partial protection against DM-associated fibrosis after Nrf2 knockdown *in vivo*. **(A)** Masson and PAS staining of kidneys (*n* = 6); **(B)** calculated positive area of Masson staining (*n* = 6); **(C** and **D)** mRNA level of collagen I and III (*n* = 6); **(E)** representative blots of TGF-β, p-Smad1/5, Smad2, and GAPDH (*n* = 6); **(F**–**H)** calculated protein expression levels (*n* = 6). *p < 0.05 vs. the Con group, *^#^* *p* < 0.05 vs. the STZ group.

After knockdown of Nrf2 by lentivirus shRNA, we observed more severe DM-associated kidney injuries and dysfunction, as shown by the significant increase in the glomerular area and KW/BW and KW/TB ratios compared with the Con or shRNA group (**Figure 5J**). The results showed a 4.95-fold increase of UACR for Nrf2-silenced diabetic mice and 3.38-fold increase for wild-type diabetic mice when compared with the Con group, which means that Nrf2-silencing worsened DM-associated kidney injuries and dysfunction. Although Nrf2 knockdown worsened renal injuries and dysfunction, no animals were lost from any of experimental groups prior to the 6 months end point. What surprised us was that Myr, even after Nrf2 knockdown, could still attenuate DM-associated kidney injuries and dysfunction. This was demonstrated by decreased glomerular area, KW/BW and KW/TB ratios, and UACR compared with the siRNA + STZ group (**Figure 5**). We also analyzed kidney fibrosis after Nrf2 knockdown, and the results showed that STZ-induced DM caused visible fibrosis, increased glycogen deposition, TGF-β/Smad pathway activation, and the secretion of collagen I and collagen III, all of which were alleviated by Myr treatment after Nrf2 knockdown (**Figure 6A–H**).

### Myricetin Regulated IκBα/nuclear Factor-κB Signaling Independent on Nuclear Factor (Erythroid Derived 2)-Like 2

**Figure 7:**
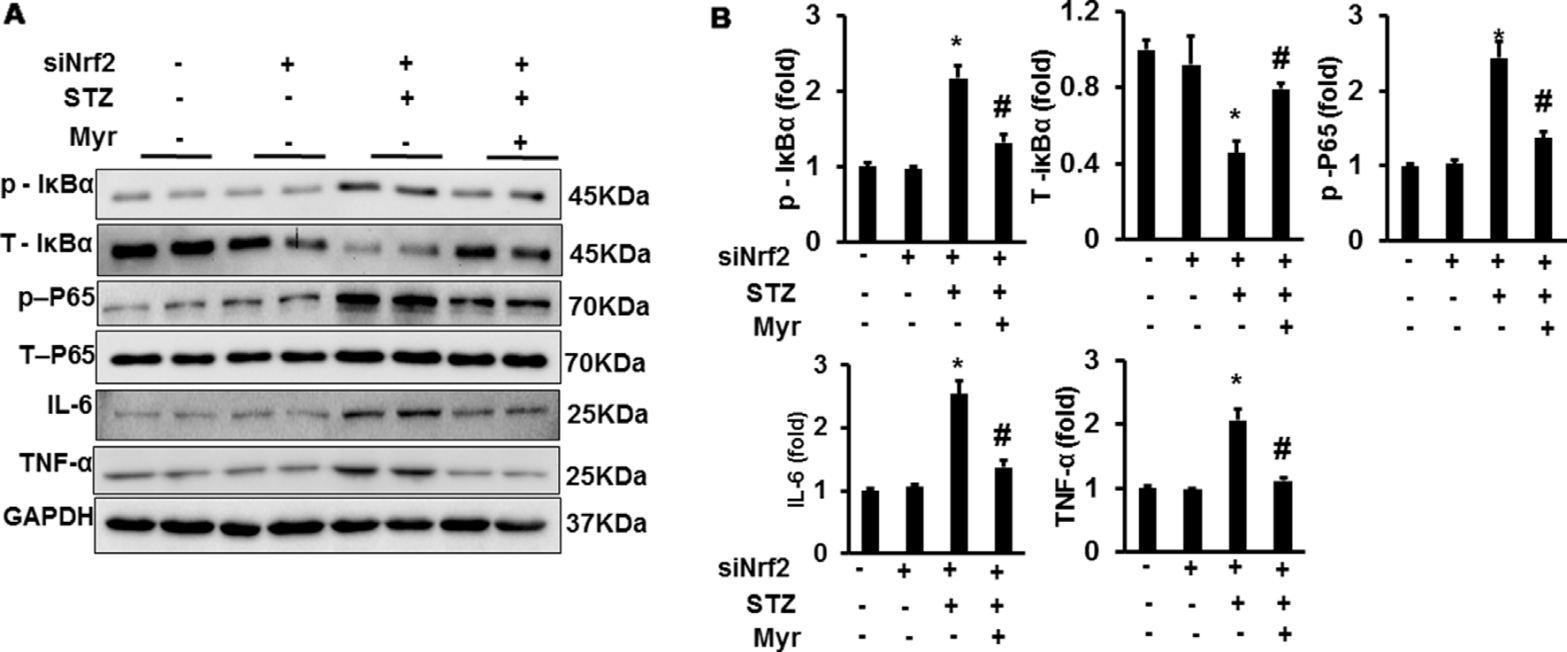
Myr regulated IκBα/NF-κB signaling independently of Nrf2. **(A)** Representative blots of p-IκB, IκB, p-P65, P65, IL-6, TNF-α, and GAPDH (*n* = 6); **(B)** calculated protein expression levels. *p < 0.05 vs. the Con group (*n* = 6), *^#^* *p* < 0.05 vs. the STZ group

After lentivirus injections, Nrf2 was significantly downregulated in mouse kidneys (**Figure 7A and B**). However, Myr treatment still markedly inhibited the expression of IL-6 and TNF-α, which are the most important inflammatory indicators of acute and chronic inflammation (**Figure 7A, C, and D**). We further examined the activity of the IκBα/NF-κB pathway. Myr significantly inhibited the phosphorylation of IκBα and NF-κB. These data indicated that Myr could inhibit inflammation *via* the IκBα/NF-κB signaling pathway being independent of Nrf2 regulation.

## Discussion

This study demonstrated that Myr attenuated DM-associated kidney injuries and dysfunction by suppressing the IκBα/NF-κB signaling pathway and promoting the expression and nuclear accumulation of Nrf2. We established a DM mouse model with five continuous injections of SZT in wild-type and Nrf2 knockdown mice, followed with or without Myr treatment for 6 months. In wild-type mice, Myr prevented Nrf2 and IκBα from degradation, which resulted in the alleviation of oxidative stress, inflammation, and fibrosis, and improvements in kidney function. However, even after Nrf2 silencing, Myr still inhibited inflammation and fibrosis, and improved kidney function.

DM-associated kidney injuries are closely related to oxidative stress and inflammation and are characterized by enlarged glomeruli and tubule-interstitial fibrosis (Ma et al., 2016; Perez-Gomez et al., 2016). Oxidative stress and inflammation are currently considered to be the main causes of the pathogenesis of DM-associated kidney injuries (Ma et al., 2016; Perez-Gomez et al., 2016). Many investigations have indicated that the inhibition of oxidative stress could alleviate diabetic complications, including DN (Zhang et al., 2016), diabetic cardiomyopathy (Wilson et al., 2018), and diabetic retinopathy (Tolentino et al., 2016). Nrf2 plays an important role in cellular resistance to oxidative stress (Al-Sawaf et al., 2015; Al-Waili et al., 2017). Under normal conditions, keap1 interacts with Nrf2 to form a complex in the cytoplasm (Al-Sawaf et al., 2015; Al-Waili et al., 2017) and acts as an inhibitor for Nrf2. Moreover, Kelch-like ECH-associated protein 1 (keap1) can also bind with Gul3-E3-ligase, thus mediating Nrf2 degradation through the ubiquitin–proteasome system and repression of transcription function (Al-Sawaf et al., 2015; Al-Waili et al., 2017). During oxidative stress, Nrf2 escapes from keap1, translocates into the nucleus, and triggers the transcription of phase II detoxification enzymes and antioxidants, such as HO-1 and NQO1, finally leading to resistant oxidative stress (Al-Sawaf et al., 2015; Al-Waili et al., 2017).

In several diabetes-associated animal models, increased expression of Nrf2 is observed in the first 3 months but decreased expression is seen after 3 to 6 months (Cui et al., 2012; Bai et al., 2013). Consistent with previous studies, we also found a significant decrease of Nrf2 in this study after 6 months of STZ treatment. At least two mechanisms could explain the discrepancy in the expression of Nrf2 during the development and progression of diabetes. First, it is assumed that Nrf2 is quickly upregulated at an early stage as an adaptive mechanism but then downregulated in cells or tissues at the late stage because of exorbitant or long-lasting oxidative stress. Thus, cardiac or kidney damage would be more evident without the adaptive upregulation with Nrf2 deficiency (Zheng et al., 2011; Zhang et al., 2018). Second, multiple signaling pathways are activated in diabetes. These signaling pathways inhibit the degradation or contribute to the overproduction of keap1, which promotes Nrf2 ubiquitination, and the subsequent degradation results in Nrf2 down-regulation. For example, excessive activation of c-Jun N-terminal kinase-induced keap1 expression results in degradation of Nrf2 under high glucose or diabetic conditions (Zhang et al., 2018).

In the STZ-induced diabetes mouse model, mice with Nrf2 knockout show increased production of ROS and severe oxidative DNA damage (Jiang et al., 2010). In contrast, inhibition of the degradation and promotion of nuclear accumulation of Nrf2 have been demonstrated to protect against diabetes-associated kidney injury (Al-Sawaf et al., 2015; Al-Waili et al., 2017). MG132, a proteasome inhibitor, has shown preventive and therapeutic effects in the development and progression of DN in basic research (Cui et al., 2013; Kong et al., 2017). In clinical research, bardoxolone methyl was the first drug to markedly increase glomerular filtration rate in a cohort of 227 chronic kidney disease patients (Pergola et al., 2011). These proteasome inhibitors share the common characteristic of reducing Nrf2 ubiquitination, decreasing its degradation, and promoting nuclear translocation (Pergola et al., 2011; Cui et al., 2013; Kong et al., 2017). Consistent with our findings in this study, Myr promoted the expression of Nrf2 and nuclear accumulation. This partially explained why Myr treatment could attenuate DM-associated kidney injury. In previous published data, Myr has been suggested to specifically regulate Nrf2 expression (Qin et al., 2013; Xia et al., 2016; Liao et al., 2017). According to the published investigations, Myr is suggested to have a weaker effect in boosting the expression of Nrf2 (Qin et al., 2013; Liao et al., 2017). However, it should be noted that some studies have reported a detrimental effect of continuous over-stimulation of Nrf2 in DN. Therefore, moderate activation of Nrf2 might be a better choice.

Besides antioxidative stress function, Myr is also well known for its anti-inflammatory and other biological functions. Inflammation is crucial for the initiation and progression of DM-associated kidney injuries. NF-κB is a protein complex that manipulates a variety of genes involved in inflammation. Under physiological conditions, IκB sequesters NF-κB in the cytoplasm. However, when IκB is ubiquitinated for degradation, NF-κB can be released and translocate into the nucleus where it triggers the expression of pro-inflammatory associated genes including IL-1β, IL-6, and TNF-α. In this study, under normal conditions, Myr could inhibit the phosphorylation and degradation of IκB, thus preventing NF-κB translocation into the nucleus and expression of IL-1β, IL-6, and TNF-α. Moreover, after knockdown of Nrf2, we observed that Myr possessed anti-inflammatory effects independent of Nrf2. Previous investigations have indicated that polyphenols have a potential capability for regulating ubiquitination (Wang et al., 2015). It has been demonstrated that ubiquitination of IκBα results in the degradation of IκBα and the release of NF-κB from the resting state in the cytoplasm (Liu et al., 2012). In this study, we detected the degradation of IκBα and the release of NF-κB. The underlying mechanism might be associated with the regulation of IκBα ubiquitination, but more experimental data are needed to show whether it is a direct effect or one that is achieved through another regulatory mechanism.

In this study, we showed that Myr attenuated renal fibrosis. There is a consensus that both oxidative stress and inflammation caused TGF-β/Smad pathway activation and the resulting fibrosis. As we have shown, we found that Myr depressed oxidative stress by increasing Nrf2, and inhibited inflammation by preventing IκBα phosphorylation and degradation. Thus, it is reasonable to speculate that Myr could inhibit fibrosis through the TGF-β/Smad pathway. Kandasamy and Ashokkumar (Kandasamy and Ashokkumar, 2014) suggested that Myr could attenuate rat renal fibrosis induced by STZ combined with cadmium, and the underlying mechanism might be associated with downregulation of the expression of sterol regulatory element binding proteins (Kandasamy and Ashokkumar, 2014), which induce the expression of TGF-β and vascular endothelial growth factor, and result in renal fibrosis (Kandasamy and Ashokkumar, 2014). In carbon tetrachloride-induced mouse liver fibrosis, Geng et al. (Geng et al., 2017) reported that Myr suppresses liver fibrosis by blocking the phosphorylation of Smad2, MAPK, and Akt. These investigations demonstrate that Myr might be a potential candidate for treating organ fibrosis; however, more experiments are needed to clarify its function and the underlying mechanism that regulates fibrosis.

## Conclusion

In summary, this investigation suggested for the first time that Myr alleviated DM-associated kidney injuries and dysfunction by inducing the expression and translocation of Nrf2 and inhibiting the IκBα/NF-κB pathway. Moreover, we found that the inhibitory effect of Myr on the IκBα/NF-κB pathway was independent of Nrf2. We suggested that polyphenol compounds, like Myr, might be promising for the treatment of DM-associated kidney injuries and dysfunction, considering their dual target effects on both oxidative stress and inflammatory pathways. Moreover, Myr is found in food (Hertog et al., 1993) and will be available and convenient for drug development.

## Author Contributions

LT and ZY contributed to the conception, experiment design, and manuscript writing; HW and YW managed animals and established the STZ-induced diabetic model; ZZ and LW carried out the experiments; LL and CZ analyzed data.

## Funding

This work was supported by a grant for Young Scholars from the first Affiliated Hospital of Zhengzhou University and by a grant of the Key Project of Medical Science and Technology from Henan Province (201501010).

## Conflict of Interest Statement

The authors declare that the research was conducted in the absence of any commercial or financial relationships that could be construed as a potential conflict of interest.
