# Supplementary material for: Myricetin Attenuated Diabetes-Associated Kidney Injuries and Dysfunction via Regulating Nuclear Factor (Erythroid Derived 2)-Like 2 and Nuclear Factor-κB Signaling
Source: Front Pharmacol. 2019 Jun 11;10:647. doi: 10.3389/fphar.2019.00647 (PMC6580432; doi:10.3389/fphar.2019.00647)
Supplement: Supplementary file 1 [file Table_1.docx]

Supplementary Material

# Supplementary Table 1

| Genes | Forward primer | Reverse primer |
| --- | --- | --- |
| CAT | GCCAATGGCAATTACCCGTC | GAGGCCAAACCTTGGTCAGA |
| SOD1 | GGAACCATCCACTTCGAGCA | CCCATGCTGGCCTTCAGTTA |
| SOD2 | CCACACATTAACGCGCAGAT | TCGGTGGCGTTGAGATTGTT |
| NQO1 | GCCCAGATATTGTGGCCGAA | AGCACTCTCTCAAACCAGCC |
| IL-1β | AACCTTTGACCTGGGCTGTC | TGATACTGCCTGCCTGAAGC |
| IL-6 | AACGATGATGCACTTGCAGA | TGTGACTCCAGCTTATCTCTTG |
| TNF-α | CCCTCACACTCACAAACCAC | ACAAGGTACAACCCATCGGC |
| Collagen I | GTGCTCCTGGTATTGCTGGT | TGTTACCCTTGGGACCTGGA |
| Collagen III | GCTGGAAAGGATGGAGAGTCA | GCGTCCATCAAAGCCTCTGT |
| TGF-β | GTCCAAACTAAGGCTCGCCA | GTTGTACAAAGCGAGCACCG |
| GAPDH | TGGTGAAGCAGGCATCTGAG | GTTGCTGTTGAAGTCGCAGG |

# CAT: catalase, SOD: superoxide dismutase 1, NQO1: NAD(P)H quinone dehydrogenase 1, IL: interleukin, TNFα: tumor necrosis factor alpha, TGFβ: transforming growth factor beta, GAPDH: glyceraldehyde-3-phosphate dehydrogenase

# Supplementary Table 2

|  | CON | Myr | STZ | STZ+Myr |
| --- | --- | --- | --- | --- |
| BG（mmol/L) | 6.1±0.45 | 5.7±0.57 | 27.3±1.26 | 27.9±1.74 |
| UACR (μg/mg) | 22.57±1.22 | 21.77±1.37 | 21.33±1.73 | 22.51±1.59 |
| BW (mg) | 24.9±0.39 | 24.8±0.53 | 25.3±0.42 | 25.2±0.33 |

## BG: blood glucose, UACR: urinary albumin to creatinine ratio, BW: body weight

## Supplementary Figures


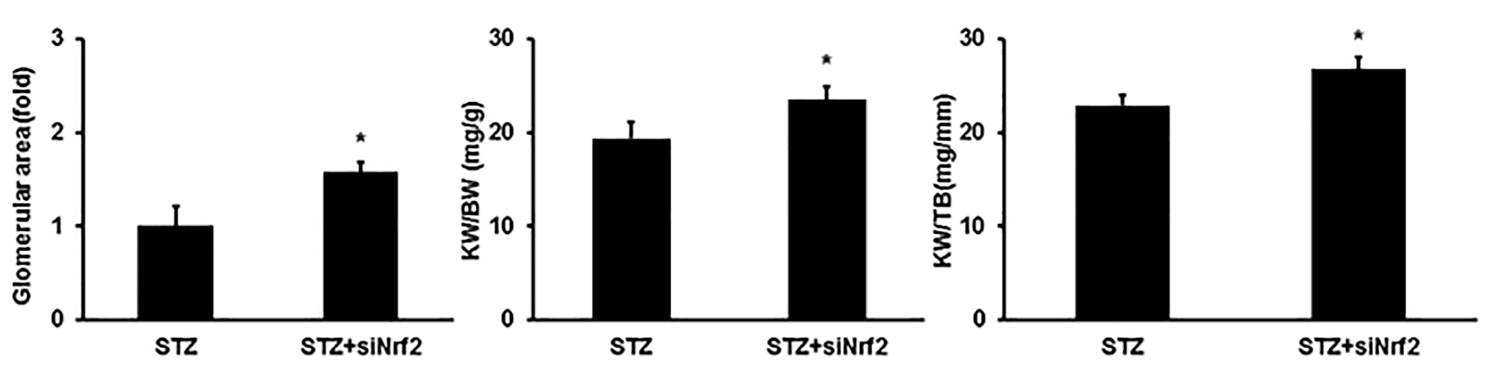


**Supplementary Figure 1.** **Silence of Nrf2 exacerbated STZ induced Morphological change of mouse kidney** KW/BW: Kidney weight/body weight, KW/TB: Kidney weight/tibia length, STZ: streptozotocin, siNRF2: silence of Nuclear factor (erythroid-derived 2)-like 2, * *p<0.05*, compared with STZ group.
